# Noninvasive Imaging OX40^+^ Activated T Cells Provides Early Warning of Rheumatoid Arthritis

**DOI:** 10.1007/s11307-023-01819-4

**Published:** 2023-04-03

**Authors:** Gang Wen, Hongwei Lei, Baochang Qi, Shao Duan, Zunyu Xiao, Chaozhe Han, Yifei Xia, Chengwei Jing, Jianyu Liu, Chao Li

**Affiliations:** 1 Department of Orthopedics, The Second Affiliated Hospital of Harbin Medical University, Harbin, 150086 China; 2 Department of Rheumatology and Immunology, The Second Affiliated Hospital of Harbin Medical University, Harbin, 150086 China; 3 Molecular Imaging Research Center of Harbin Medical University, Harbin, 150001 China

**Keywords:** OX40 (tumor necrosis factor receptor superfamily, member 4), Rheumatoid arthritis, NIRF imaging, Activated T cell, Antibody

## Abstract

**Purpose:**

The goal of this study was to develop an imaging probe—IRDye-680RD-OX40 mAb—that can be used for noninvasive imaging and optical imaging of rheumatoid arthritis (RA). OX40/OX40 ligand (OX40L) interactions have been shown to exert potent costimulatory effects on T cell activation. Detectable change in T cell activation profiles was observed in early RA.

**Methods:**

OX40 expression pattern was analyzed by flow cytometry. N-hydroxysuccinimide (NHS) esters are used to label proteins selectively on free amino groups of OX40 monoclonal antibody (mAb). Characterization of IRDye-680RD-OX40 mAb was measured and a fluorescence spectrum gathered. Cell binding assay was also performed between activated and naïve murine T cells. Longitudinal near-infrared fluorescence (NIRF) imaging of the probe was performed on day 8, day 9, day 10, and day 11 of adjuvant-induced arthritis (AIA) mouse model. Paw thickness and body weight were compared between the OX40 mAb and IgG injection groups.

**Results:**

NIRF imaging with IRDye-680RD-OX40 mAb revealed strong OX40-positive responses with high specificity. Flow analysis showed that OX40 was specifically expressed on the surface of T cells in RP and spleen of AIA model. The AIA group was significantly differentiated from the control group at all time points with imaging monitoring. The region of interest (ROI) was in line with ex vivo imaging and biodistribution study. This study highlights the potential utility of the OX40 NIRF imaging as a new strategy for RA prediction and T cell monitoring.

**Conclusion:**

The results provide evidence that IRDye-680RD-OX40 mAb detects organized T cells activation in early RA. The optical probe was capable of detection of RA pathogenesis. It identified transcriptional responses to RA that mediate its immune functions. Thus, it may be an ideal probe for RA imaging.

**Graphical Abstract:**

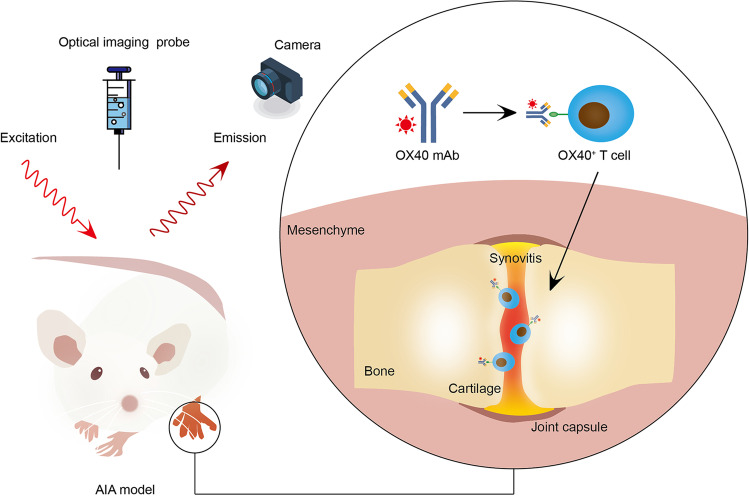

## Introduction

RA is one of the most prevalent autoimmune diseases characterized by chronic inflammation and progressive bone destruction [1]. It is well documented that early diagnosis and timely intervention could be a crucial approach for optimizing clinical patient management [2]. Standards based on the symptoms of musculoskeletal pain, swelling, and stiffness often fail in RA early diagnosis [3]. While anti-cyclic citrullinated peptide (anti-CCP), on the other hand, is considered as a specific biomarker of RA; however, the sensitivity of CCP blood testing is only 70–80%, and a negative result cannot exclude the disease [4]. Thus, developing novel diagnostic tool for RA early detection is still needed.

According to previous studies [5], activated T cells are considered as the major mediators of RA occurrence, which were involved in the pathogenesis of RA from the very beginning across the whole disease progression [6]. Thus, imaging activated T cells may be an ideal way to achieve this goal [7]. In a previous study, [18F]F-AraG was employed as a PET imaging agent for detecting T cell activation within inflammatory arthritis [8]. However, despite the activated T cell portions, [18F]F-AraG was also accumulated in bone marrow derived macrophages (BMDMs) and dendritic cells (DCs) due to its association with mitochondrial metabolism [9, 10]. Given the low specificity of [18F]F-AraG in this setting, our group had focused on imaging T cell surface activation biomarkers. In our recent published study, we had developed an optical imaging probe targeting 4-1BB, which enables caption of both 4-1BB^+^ CD4^+^ and CD8^+^ activated T cells *in vivo* with high specificity and sensitivity [11]. Based on the quantitation of NIRF imaging data, we could precisely distinguish AIA mouse from the control group, which demonstrated great potential of imaging activated T cells for optimizing RA diagnosis.

While from our subsequent RNAseq and flow cytometry data, we realized that activated CD4^+^ T cells are the primary portion which drives RA pathogenesis. OX40 is a T cell costimulatory molecule restricted to activated CD4^+^ T cells [12]. Interaction between OX40 and its ligand OX40L plays an important role in T cell activation, expansion, and survival, which is thought to be one of the predominant driven factors in RA pathogenesis [13]. Previous studies had reported the utility of OX40-immunoPET in acute graft-versus-host disease (aGvHD) detection and therapeutic response prediction of cancer immunotherapy [14, 15]. In the current study, we hypothesize that whether OX40 could be used in RA detection. Flow cytometry study was employed for analysis of OX40 expression in an AIA mouse model. For IRDye-680RD-OX40 mAb synthesis, anti-OX40 mAb was conjugated to IRDye680 optical dye via NHS ester amine reaction. The specificity of IRDye-680RD-OX40 mAb targeting OX40^+^ activated T cells was tested by both ex vivo cell binding assay and *in vivo* NIRF imaging studies. The robust data demonstrated that we had developed a novel optical imaging probe IRDye-680RD-OX40 mAb for OX40^+^ activated T cells tracking, and OX40 NIRF imaging was a promising strategy for RA early detection.

## Materials and Methods

### General Reagents

Complete Freund’s adjuvant (CFA) is provided by Chondrex Company (Woodinville, USA). PE anti-mouse CD4, FITC anti-mouse CD8a, and PeCy7 anti-mouse OX40 antibody were supplied by BioLegend (San Diego, CA). InVivo MAb anti-mouse OX40 (clone: OX-86) and InVivoPlus rat IgG2a isotype control (clone: 2A3) were supplied by BioXCell (West Lebanon, USA). IRDye® 680RD NHS Ester Infrared Dye was supplied by Li-cor (Lincoln, USA). Fixable Viability Stain 780 was purchased from BD Pharmingen (San Diego, USA). Dimethyl sulfoxide (DMSO) Hybri-Max (TM) sterile-filtered, BioReagent was purchased from Sigma (St. Louis, MO, USA). AbC™ total antibody compensation bead kit was acquired from Thermo Fisher (Waltham, MA, USA). Paraformaldehyde, 4% and phosphate buffered solution (PBS) was supplied by Solarbio (Beijing, China). Fetal bovine serum (FBS) was purchased from Invitrogen (New York, America).

### AIA Model

All animal studies were carried out in accordance with protocols approved by the Institutional Animal Care and Use Committee of Harbin Medical University’s Second Affiliated Hospital. For AIA model generation, 8–10 weeks male BALB/c mice (20–25 g) were given a single injection of 0.5 mL/kg CFA into the plantar subcutaneous tissue of RP, whereas equal amount of PBS was injected into RP of control mice [8]. As a control, equal amounts of PBS were injected subcutaneously into each animal’s left hind paw (LP). Animals were monitored every day after injection. Paw thickness was considered as the measurements of paw swelling related to arthritis. A caliper was used to gauge paw thickness.

### Flow Cytometry

On day 11 after CFA injection, RP and spleen were collected. Spleens were cleaned and fat was removed before passing through a 40 µm filter with FACS buffer (PBS containing 2% FBS). The preceding approach was followed to create a single cell suspension [16]. The following antibodies were used in FACS staining: PE anti-mouse CD4, FITC anti-mouse CD8a, and PeCy7 anti-mouse OX40. Data was collected using a BD FACSCanto II Flow Cytometer, and version 10.7.1 of FlowJo was used for analysis.

### Histological Analysis

For HE staining, RP was harvested on day 7, fixed with 4% paraformaldehyde for 24 h, and decalcified with 10% ETDA for 20 days, then embedded in paraffin and cut into sections of 5 μm thick. To determine infiltration of immune cells from both the AIA and control groups, every paw section was stained with hematoxylin and eosin (H&E; Solarbio, Beijing, China). Specimens were observed under a BX53 microscope (Olympus, Tokyo, Japan) and measured with Software (Image J).

### Synthesis and Characterization of IRDye-680RD-OX40 mAb, T Cell Isolation, and Cell Uptake Study

The IRDye-680RD NHS ester was dissolved in DMSO to a final concentration of 5 mg/mL. OX40 mAb was diluted in sterile PBS to 1 mg/mL. IRDye-680RD NHS ester was mixed with OX40 mAb, at a molar ratio of OX40 mAb to IRDye-680RD of 1:10. The mixture was incubated at 4 °C overnight. For purification, a Vivaspin2 50 KDa cutoff MWCO spin filter (GE Healthcare, Piscataway, USA) was used. The NanoDrop 2000 UV–vis spectrophotometer (Thermo Fisher, Waltham, MA, USA) was used to determine the final concentration of IRDye-680RD-OX40 mAb.

BALB/c mice were anesthetized with 2% isoflurane. Spleens were collected immediately after euthanasia and smashed through a 40 μm cell strainer. T cells were isolated using the EasySep Mouse T cell Isolation Kit’s standard protocol (STEMCELL Technologies, Canada) and incubated in Iscove’s Modified Dulbecco’s Medium (IMDM, Thermo Fisher) with Cell Stimulation Cocktail (Thermo Fisher) at 37 °C. Both resting and activated T cells were collected, counted, and incubated with IRDye-680RD-OX40 mAb for 1 h at 37 °C 72 h later. All samples were analyzed using a BD flow cytometer after three washes with sterile PBS.

### *In Vivo* and *Ex Vivo* NIRF Imaging

Bruker InVivo FX PRO *in vivo* fluorescence imaging was performed and analyzed using Bruker Molecular Imaging Software (IB5438150 Rev. B 12/12, Bruker, USA). On day 7, mice were anesthetized with 2% isoflurane and injected with 20 μg IRDye-680RD-OX40 mAb via tail vein. Images were taken on days 8, 9, 10, and 11 using the following settings: (f-stop 2.5, FOV 200 mm, 750 nm WA Emission Filter, 72 mm, 670 nm Excitation Filter, 25 mm). After the final scan, mice were euthanized, and organs (heart, liver, lung, spleen, kidney, right paw, left paw, femur, muscle, and intestine) were collected and imaged under the same conditions. Bruker Molecular Imaging Software was used to analyze all of the data. The quantification was normalized as p/sec/cm2/sr.

### Statistical Analysis

The PRISM 9 platform was used for all data analysis (GraphPad). Data analysis methods included unpaired 2-tailed Student’s *t*-tests and one- or two-way analyses of variance (ANOVA). Statistics were considered significant for *P* values under 0.05.

## Results

### Establishment of Adjuvant-Induced Arthritis Mouse Model and Safety Assessment of OX40 mAb Injection

**Fig. 1:**
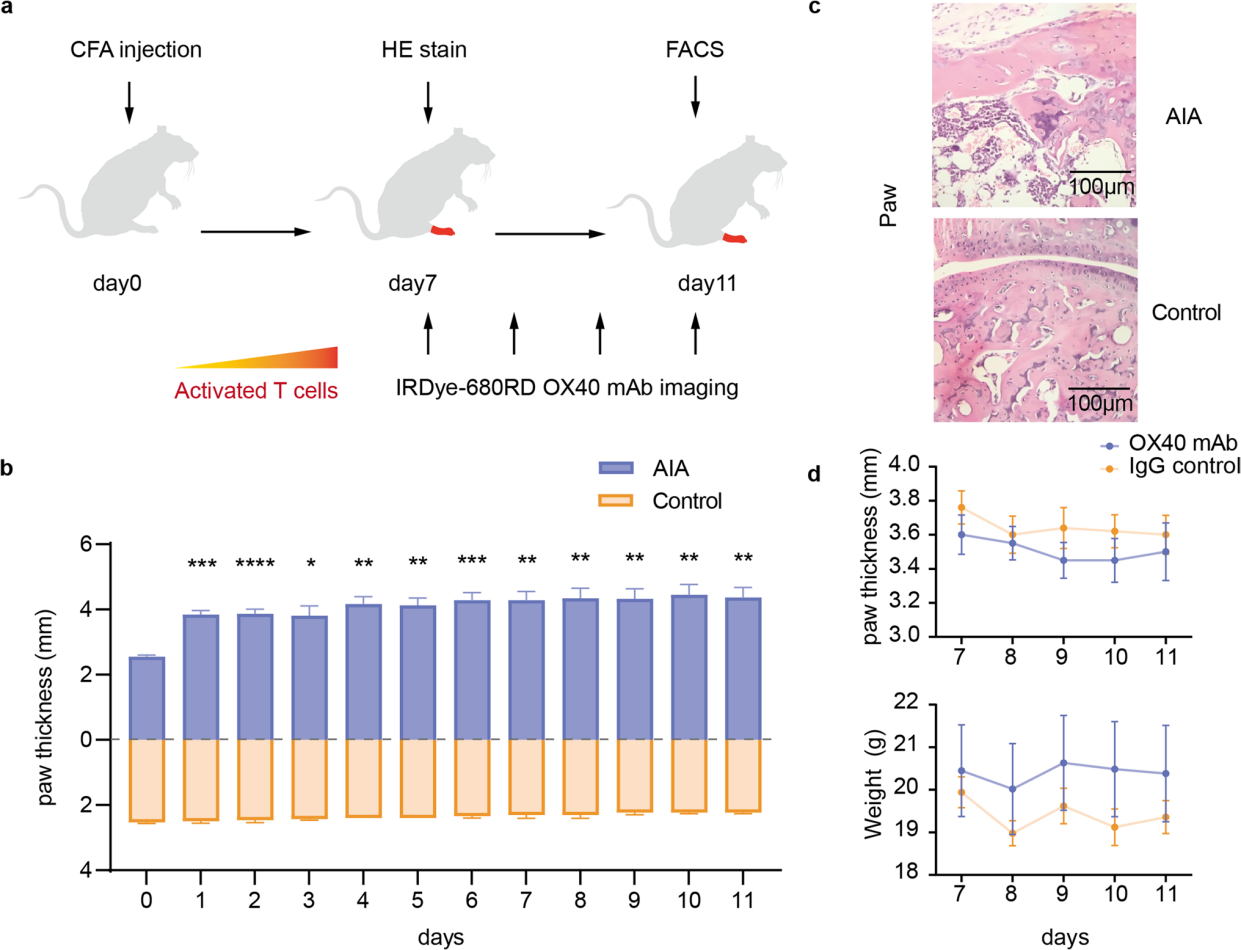
AIA mouse model establishment and safety assessment of OX40 mAb injection. **a** Scheme of animal establishment and NIRF imaging studies. **b** The measurements of paw thickness after mouse model establishment. **c** HE staining of ankle joint of the AIA and control groups. **d** Safety assessment of AIA models receiving equal amount isotype control IgG or OX40 mAb. All values represent the mean ± SEM unless otherwise specified. Unpaired 2-tailed Student’s *t*-test was used for analyses, ****, *P* < 0.0001; ***, *P* < 0.001; **, *P* < 0.01; *, *P* < 0.05

Our conceptual study design is described in sketch (Fig. 1a). Inflammatory arthritis model was induced in healthy male BALB/c mice (*n* = 7) by injecting CFA into right hind paw (RP) on day 0, whereas the control groups were injected with PBS (*n* = 4). To monitor the severity of arthritis, paw thickness was measured with a caliper every day since the initial injection, and the AIA group was thicker at all time points measured, which was in agreement with previous studies (Fig. 1b) [11]. To further validate the inflammatory microenvironment generated by CFA injection, HE staining was performed at day 7 with RP tissues from both the AIA and control groups, massive infiltration of inflammatory cells could be observed in the AIA group (Fig. 1c). The engineering strategies determined the effectiveness, reliability, and safety of the molecular imaging probes [17]. It had been reported that OX40 mAb (clone: OX-86) we employed may exacerbate aGvHD disease [8], thus before *in vivo* NIRF imaging study, we had tested whether injection of OX40 mAb would cause additional toxicity. On day 7, 20 μg of unconjugated OX40 mAb or IgG isotype control were injected into AIA mouse, the changes in paw thickness and body weight were monitored. There was no obvious difference in body weight and paw thickness (*P* > 0.05) between two cohorts, which indicated the safety of OX40 mAb injection (Fig. 1d).

### OX40 Was an Indicator of T Cell-Mediated Immune Response in RA

**Fig. 2:**
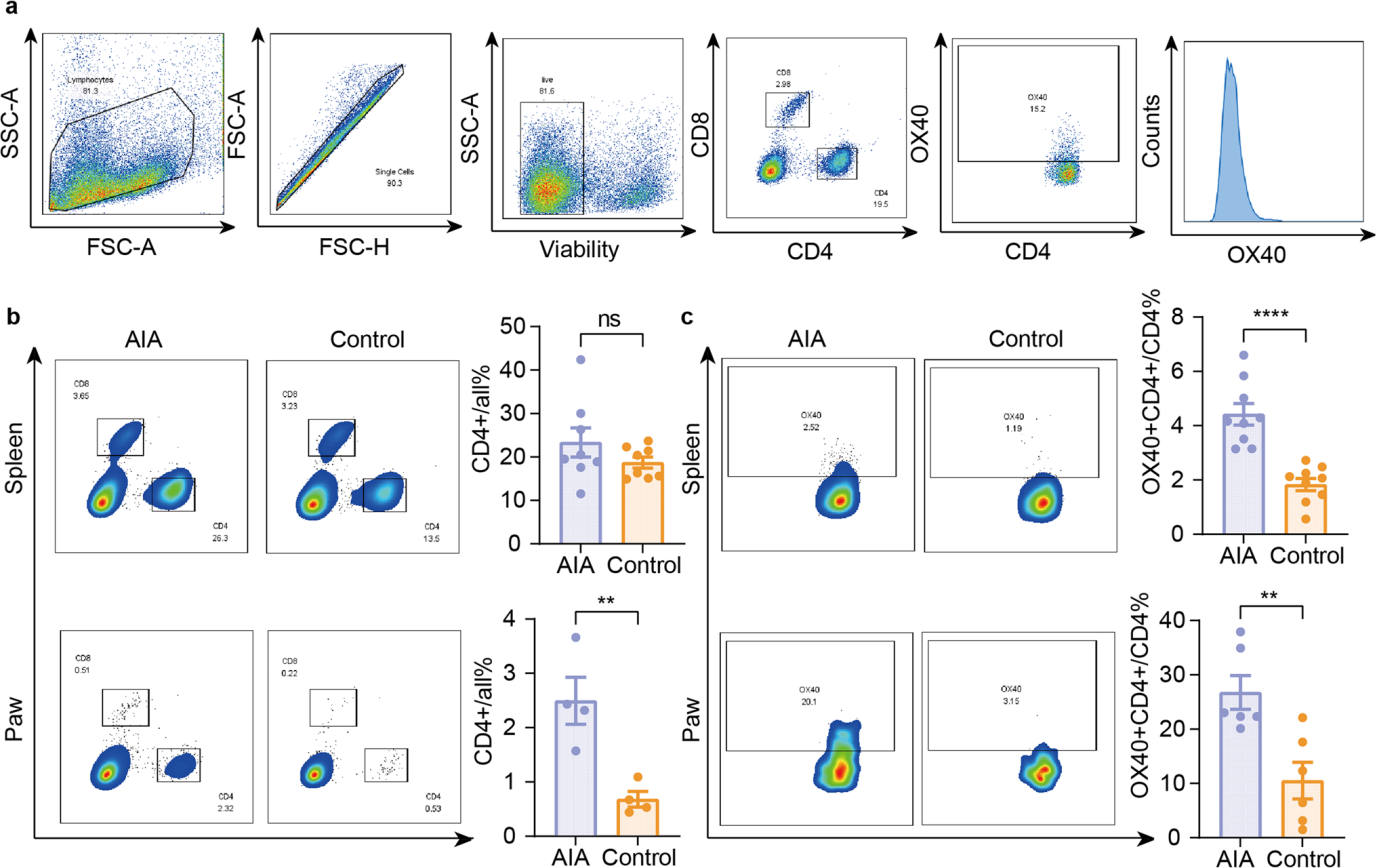
FACS analysis of OX40 expression in AIA mouse model. **a** Gating strategy illustrating cell populations sub-gated to the level of mouse OX40 + CD4 + activated T cells; **b** comparison of CD4 + T cells in spleen and right hind paw between the AIA and control groups; **c** comparison of OX40 expression in spleen and right hind paw between the AIA and control groups. All values represent the mean ± SEM unless otherwise specified. Unpaired 2-tailed Student’s *t*-test was used for analyses, ****, *P* < 0.0001; ***, *P* < 0.001; **, *P* < 0.01; *, *P* < 0.05

To validate whether OX40 could be an indicator of T cell-mediated immune response during RA pathogenesis, flow cytometry study was performed between the AIA and control groups. Gating strategy illustrating cell portions to the level of murine OX40^+^CD4^+^ T cells is shown in Fig. 2a. Massive infiltration of CD4^+^ T cells was observed in RP tissue from the AIA group, compared to control mice (*P* < 0.01), whereas no difference was detected in spleen (Fig. 2b). As we expected, after CFA injection, higher percentage of OX40^+^CD4^+^ T cells were detected in RP from AIA mice (*P* = 0.0053); moreover, increased OX40^+^CD4^+^ T cells were also observed in spleen from the AIA group (*P* < 0.0001), which demonstrated systemic immune response triggered by CFA injection (Fig. 2c).

### *In Vitro* Cell Binding Assay and *In Vivo* NIRF Imaging Stud*y*

**Fig. 3:**
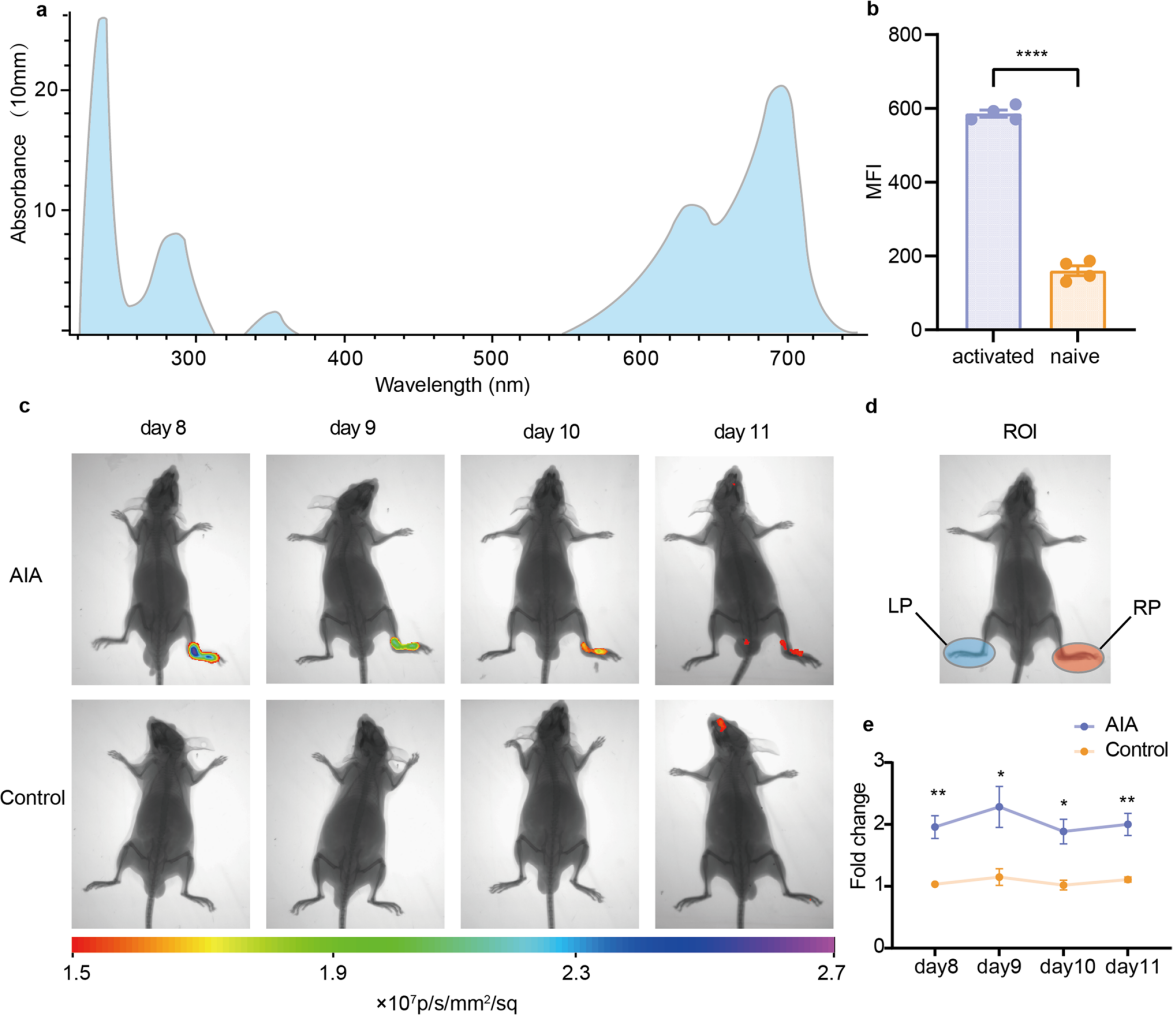
*In vitro* cell binding assay and *in vivo* NIRF imaging. **a** Cell uptake of IRDye-680RD-OX40 mAb in activated and naivë T cells. Activated group enhanced IRDye-680RD-OX40 mAb uptake notably. **b** Characterization of IRDye-680RD-OX40 mAb via spectrum analysis (*P* < 0.0001). **c** *In vivo* NIRF imaging from day 8 to day 11. **d** ROI atlas of RP and LP. **e** Quantification of RP to LP ratio at all time points examined. All values represent the mean ± SEM unless otherwise specified. Unpaired 2-tailed Student’s *t*-test was used for analyses, ****, *P* < 0.0001; ***, *P* < 0.001; **, *P* < 0.01; *, *P* < 0.05

We synthesized OX40-targeting NIRF imaging agent—IRDye-680RD-OX40 mAb—via OX40 mAb and NHS ester crosslinking, then purified through Vivaspin2 50 K cutoff spin filter, according to previous protocols [11]. After synthesizing, the absorbance of the probe was measured by nanodrop, which displayed an absorbance maximum of 690 nm (Fig. 3a). For *in vitro* cell binding assay, 2 μg of IRDye-680RD-OX40 mAb was incubated with activated and naive T cells at 37 °C for 1 h to confirm the specificity of IRDye680RD-OX40 mAb to activated T cells. Using a flow cytometer, the median fluorescence intensity (MFI) was estimated. The MFI of the activated T cell group (586 ± 9.94) was 3.65-fold higher than that in the naive group (160.5 ± 13.49) (*P* < 0.0001) (Fig. 3b). For NIRF imaging study, 20 μg of IRDye-680RD-OX40 mAb was administrated via tail vein at day 7, images were acquired from day 8 to day 11. There were visually detectable higher signals in RP from the AIA group (Fig. 3c). To quantify the NIRF signals, ROIs were drawn, and the fluorescence intensity ratio between RP and LP was used to compare the ROI profiles of the AIA and control groups (Fig. 3d). RP/LP ratio quantification in the AIA group was significantly higher than that in the control group (day 8: AIA = 1.958 ± 0.184, control = 1.033 ± 0.047; day 9: AIA = 2.283 ± 0.331, control = 1.150 ± 0.135; day 10: AIA = 1.886 ± 0.199, control = 1.020 ± 0.079; day 11: AIA = 2.000 ± 0.178, control = 1.110 ± 0.040 and *P* value = 0.005035, 0.035668, 0.010801, and 0.005248, respectively) (Fig. 3e). This finding may be attributed to the increased infiltration of OX40^+^ CD4^+^ activated T cells in RP of the AIA group, which is consistent with the flow cytometry data presented above.

### *Ex Vivo* Biodistribution and OX40 NIRF Imaging for Predicting T Cell Response in RA

**Fig. 4:**
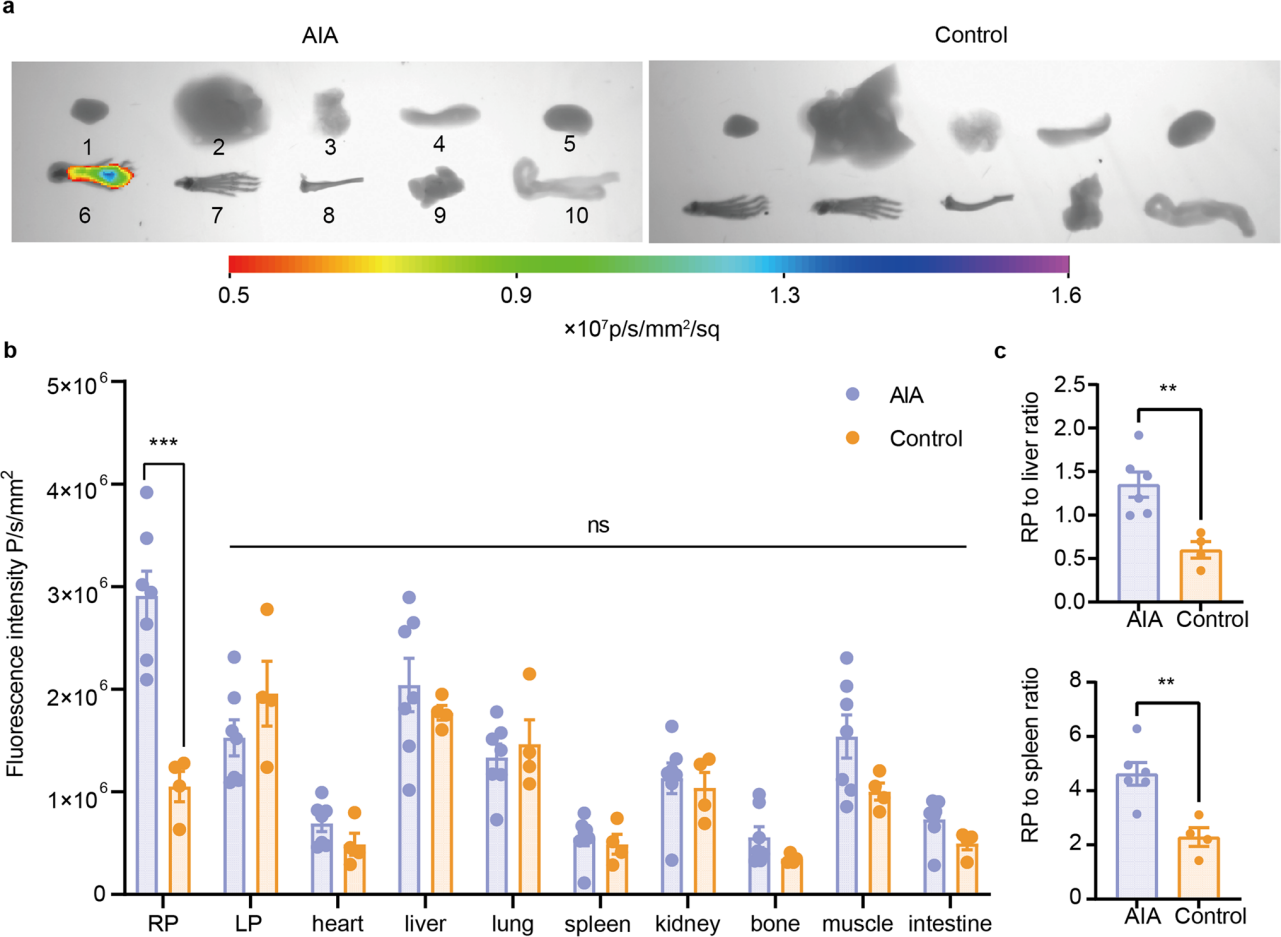
Ex vivo biodistribution of IRDye-680RD-OX40 mAb. **a** Representative ex vivo NIRF/X-ray images of organs (1. heart, 2. liver, 3. lung, 4. spleen, 5. kidney, 6. right hind paw, 7. left hind paw, 8. femur, 9. muscle, 10. intestine). **b** Semi-qualitative comparison of the fluorescence intensity. **c** RP to liver (*P* = 0.0049) and spleen (*P* = 0.0041) ratios between the AIA and control groups. All values represent the mean ± SEM unless otherwise specified. Unpaired 2-tailed Student’s *t*-test was used for analyses, ****, *P* < 0.0001; ***, *P* < 0.001; **, *P* < 0.01; *, *P* < 0.05

Ex vivo NIRF imaging was performed to validate the accuracy of *in vivo* imaging data on day 11, right after the last scan (Fig. 4a). Significant difference in fluorescence intensity of RP between two cohorts (*P* = 0.0041) could be detected, whereas no difference was found in other organs (Fig. 4b). In the AIA groups, the ratio of RP/liver and RP/spleen was 1.350 ± 0.144 and 4.623 ± 1.021 which was higher than that in the control group (0.599 ± 0.095, 2.289 ± 0.347) (*P* = 0.0049, *P* = 0.0041) (Fig. 4c). These results indicate that the IRDye-680RD-OX40 mAb NIRF signal is specifically visualizing OX40 expression within RP.

**Fig. 5:**
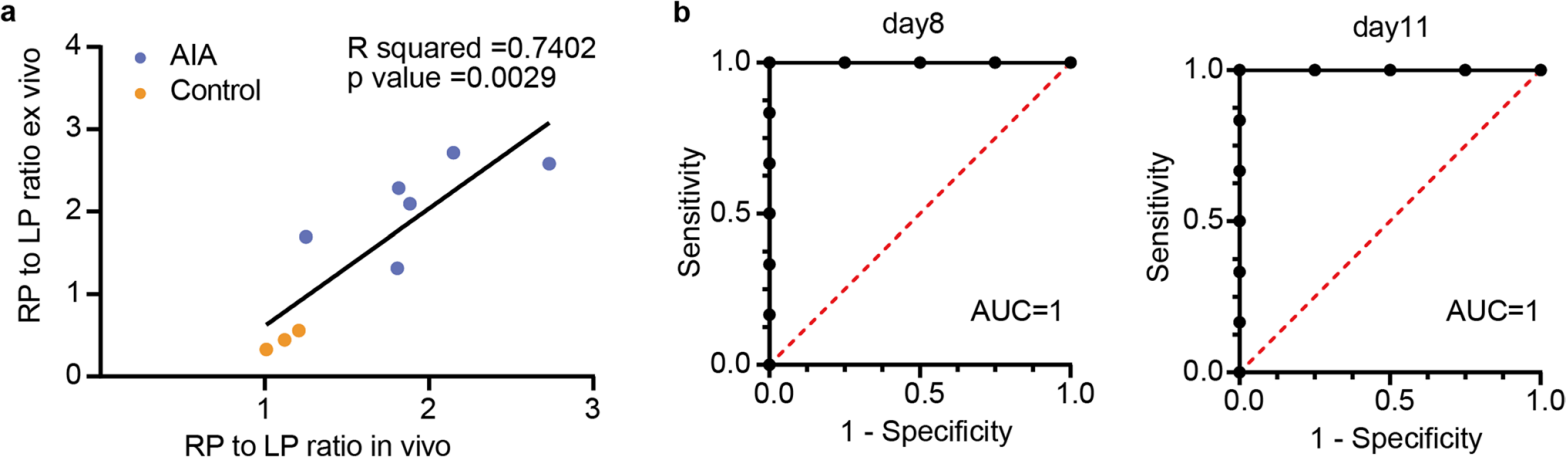
IRDye-680RD-OX40 mAb NIRF imaging provides early warning of inflammatory arthritis pathogenesis. **a** Linear regression of *in vivo* RP/AP ratio with ex vivo RP/AP ratio. **b** Receiver operating characteristic curve analysis showing diagnostic value of OX40 NIRF imaging in distinguishing AIA mouse from the control cohort

The AIA group had a higher ex vivo RP/LP ratio than the control group, which was consistent with the *in vivo* ROI measurements. Good consistency between ROI and ex vivo biodistribution quantification could be observed via linear regression (*R* square = 0.7402, *P* value = 0.0029) (Fig. 5a). Receiver operating characteristic (ROC) curves based on RP/LP fold change in the ROI at all time periods photographed could fully differentiate the AIA from the control group (area under the curve (AUC) = 1.000) (Fig. 5b), indicating the great value of OX40 NIRF imaging in detecting inflammatory arthritis pathogenesis.

## Discussion

RA is a chronic autoimmune disease marked by joint inflammation and degeneration. To avoid joint damage and physical handicap, it is crucial to diagnose and treat the disease at early stages [18]. Given the low specificity and sensitivity of CCP and RF blood testing, researchers had focused on developing noninvasive imaging approaches to achieve this goal. In the current study, we had developed a novel optical imaging probe for detecting OX40^+^CD4^+^ activated T cells, the *in vivo* imaging data demonstrated that OX40 NIRF imaging could be a reliable tool for RA early diagnosis.

Since activated T cells are considered as the primary mediators and the initial event in RA pathogenesis [19], imaging activated T cell infiltration and distribution is potentially the most sensitive way to identify the early stages of RA pathogenesis. Thus, various imaging reagents for detecting activated T cells have been developed and tested both in preclinical and clinical settings, such as small molecules targeting certain metabolic pathways, probes targeting specific T cell phenotypes, and secreted cytokines [20]. Following the costimulatory signal, which is regarded as the first signal during the development of adaptive immune response, OX40 will be quickly upregulated on the surface of T cells, prior to any metabolic pathway and cytokine secretion [21]. Thus, imaging OX40 should be the earliest events we could detect in the inflammatory microenvironment. Based on our flow cytometry data, we found that the CD4^+^ T cell is the prominent portion in RA, which is in line with previous study [22], imaging activated CD4^+^ T cell may be more practical. Unlike 4-1BB, OX40 is strictly expressed on CD4^+^ T cells [23], which makes OX40 an ideal biomarker for activated CD4^+^ T cell tracking. Thus, the differences detected between the AIA and control groups should be attributed to the upregulation of OX40 molecules in the inflammatory microenvironment.

While the OX40 imaging study we presented here does have limitations, though, the AIA model we used in this study only causes acute inflammation [24], which is a little bit different from the chronic process of RA pathogenesis. To improve our work in the future, it is necessary to evaluate mouse models that can better mimic the microenvironment of rheumatoid arthritis, and furthermore, in consideration of clinical translation, a humanized mouse model warrants evaluation. The optical dye IRDye680 we employed belongs to NIR-I (700–900 nm), with the advantage of non-radioactive, convenient, easy preparation, and low cost NIR-I imaging is suitable for superficial disease imaging, such as RA, systemic lupus erythematosus (SLE), and melanoma [25]. But due to its poor penetration, NIR-I imaging often fails in deep tissue imaging, which limits further translation. To overcome these challenges, we intend to use NIR-II (near-infrared region-II) dye (1000–1700 nm) [26, 27] to enhance imaging quality in the following studies. A multi-modality technology, positron emission tomography (PET)/magnetic resonance (MR) may also help improve our work in the future, by combining refined bone structure with biological information of OX40^+^ CD4^+^ T cells in inflammatory arthritis microenvironment.

## Conclusions

We had identified OX40 as a robust biomarker for identifying activated CD4^+^ T cells in RA, and OX40 NIRF imaging enabled early warning of RA with high specificity and sensitivity *in vivo*. Based on the data presented here, we believe that further humanized OX40 imaging probes warrant synthesis and assessment.

## Data Availability

The data needed to evaluate the conclusions of the paper are present in the paper.

## Abbreviations

*RA*: Rheumatoid arthritis
*AIA*: Adjuvant-induced arthritis
*mAb*: Monoclonal antibody
*NIRF*: Longitudinal near-infrared fluorescence
*anti-CCP*: Anti-cyclic citrullinated peptide
*BMDMs*: Bone marrow derived macrophages
*DCs*: Dendritic cells
*aGvHD*: Acute graft-versus-host disease
*CFA*: Complete Freund’s adjuvant
*PBS*: Phosphate buffered solution
*FBS*: Fetal bovine serum
*LP*: Left paw
*RP*: Right hind paw
*MFI*: Average fluorescence intensity
*AUC*: Area under the curve
*SLE*: Systemic lupus erythematosus
*NIR-II*: Near-infrared region-II
*PET*: Positron emission tomography
*MR*: Magnetic resonance

## References

[1] He X, Liu J, Liang C, Badshah SA, Zheng K, Dang L, Guo B, Li D, Lu C, Guo Q, Fan D, Bian Y, Feng H, Xiao L, Pan X, Xiao C, Zhang B, Zhang G, Lu A (2019). Osteoblastic PLEKHO1 contributes to joint inflammation in rheumatoid arthritis. EBioMedicine.

[2] Marcusa DP, Mandl LA (2014). Challenges in imaging in preclinical rheumatoid arthritis. Rheum Dis Clin North Am.

[3] Sparks JA (2019). Rheumatoid arthritis. Ann Intern Med..

[4] Niewold TB, Harrison MJ, Paget SA (2007). Anti-CCP antibody testing as a diagnostic and prognostic tool in rheumatoid arthritis. QJM.

[5] Weyand CM, Wu B, Goronzy JJ (2020). The metabolic signature of T cells in rheumatoid arthritis. Curr Opin Rheumatol.

[6] Weyand CM, Goronzy JJ (2021). The immunology of rheumatoid arthritis. Nat Immunol.

[7] Li C, Han C, Duan S, Li P, Alam IS, Xiao Z (2022). Visualizing T-cell responses: the T-cell PET imaging toolbox. J Nucl Med.

[8] Franc BL, Goth S, MacKenzie J, Li X, Blecha J, Lam T, Jivan S, Hawkins RA, VanBrocklin H (2017). *In vivo* PET imaging of the activated immune environment in a small animal model of inflammatory arthritis. Mol Imaging.

[9] Levi J, Duan H, Yaghoubi S, Packiasamy J, Huynh L, Lam T, Shaikh F, Behera D, Song H, Blecha J, Jivan S, Seo Y, VanBrocklin HF (2022). Biodistribution of a mitochondrial metabolic tracer, [18F]F-AraG, in healthy volunteers. Mol Imaging.

[10] Levi J, Lam T, Goth SR, Yaghoubi S, Bates J, Ren G, Jivan S, Huynh TL, Blecha JE, Khattri R, Schmidt KF, Jennings D, VanBrocklin H (2019). Imaging of activated T cells as an early predictor of immune response to anti-PD-1 therapy. Cancer Res.

[11] Duan S, Han C, Xia Y, Jing C, Dong B, Zhang X, Wang W, Wang Y, Zhang M, Li P, Chen W, Xiao Z, Li C (2022). Fluorophore-conjugated 4–1BB antibody enables early detection of T-cell responses in inflammatory arthritis via NIRF imaging. Eur J Nucl Med Mol Imaging.

[12] Croft M (2010). Control of immunity by the TNFR-related molecule OX40 (CD134). Annu Rev Immunol.

[13] Yoshioka T, Nakajima A, Akiba H, Ishiwata T, Asano G, Yoshino S, Yagita H, Okumura K (2000) Contribution of OX40/OX40 ligand interaction to the pathogenesis of rheumatoid arthritis. Eur J Immunol 30(10):2815–2823 10.1002/1521-4141(200010)30:10<2815::AID-IMMU2815>3.0.CO;2-# 11069062

[14] Alam IS, Simonetta F, Scheller L, Mayer AT, Murty S, Vermesh O, Nobashi TW, Lohmeyer JK, Hirai T, Baker J, Lau KH, Negrin R, Gambhir SS (2020). Visualization of activated T cells by OX40-immunoPET as a strategy for diagnosis of acute graft-versus-host disease. Cancer Res.

[15] Nobashi TW, Mayer AT, Xiao Z, Chan CT, Chaney AM, James ML, Gambhir SS (2021). Whole-body PET imaging of T-cell response to glioblastoma. Clin Cancer Res.

[16] Shi C, Jia T, Mendez-Ferrer S, Hohl TM, Serbina NV, Lipuma L, Leiner I, Li MO, Frenette PS, Pamer EG (2011) Bone marrow mesenchymal stem and progenitor cells induce monocyte emigration in response to circulating toll-like receptor ligands. Immunity 34(4):590–601 10.1016/j.immuni.2011.02.016 PMC3081416 21458307

[17] Yang E, Liu Q, Huang G, Liu J, Wei W (2022). Engineering nanobodies for next-generation molecular imaging. Drug Discov Today.

[18] Zhao J, Li ZG (2018). The challenges of early diagnosis and therapeutic prediction in rheumatoid arthritis. Int J Rheum Dis.

[19] Perkins DL (1998) T-cell activation in autoimmune and inflammatory diseases. Curr Opin Nephrol Hypertens 7(3):297–303. 10.1097/00041552-199805000-00010 10.1097/00041552-199805000-00010 9617561

[20] Wei W, Jiang D, Ehlerding EB, Luo Q, Cai W (2018). Noninvasive PET imaging of T cells. Trends Cancer.

[21] Webb GJ, Hirschfield GM, Lane PJ (2016). OX40, OX40L and autoimmunity: a comprehensive review. Clin Rev Allergy Immunol.

[22] Choudhary N, Bhatt LK, Prabhavalkar KS (2018). Experimental animal models for rheumatoid arthritis. Immunopharmacol Immunotoxicol.

[23] Jiang J, Liu C, Liu M, Shen Y, Hu X, Wang Q, Wu J, Wu M, Fang Q, Zhang X (2017). OX40 signaling is involved in the autoactivation of CD4+CD28- T cells and contributes to the pathogenesis of autoimmune arthritis. Arthritis Res Ther.

[24] Ishii N, Takahashi T, Soroosh P, Sugamura K (2010). OX40-OX40 ligand interaction in T-cell-mediated immunity and immunopathology. Adv Immunol.

[25] Ma H, Xu M, Song Y, Zhang T, Yin H, Yin S (2019). Interferon-γ facilitated adjuvant-induced arthritis at early stage. Scand J Immunol.

[26] Slooter MD, Bierau K, Chan AB, Löwik CW (2015). Near infrared fluorescence imaging for early detection, monitoring and improved intervention of diseases involving the joint. Connect Tissue Res.

[27] Su Y, Yu B, Wang S, Cong H, Shen Y (2015). NIR-II bioimaging of small organic molecule. Biomaterials.

